# Acute toxicity, brine shrimp cytotoxicity, anthelmintic and relaxant potentials of fruits of *Rubus fruticosus* Agg

**DOI:** 10.1186/1472-6882-13-138

**Published:** 2013-06-18

**Authors:** Niaz Ali, Umer Aleem, Syed Wadood Ali Shah, Ismail Shah, Muhammad Junaid, Ghayour Ahmed, Waqar Ali, Mehreen Ghias

**Affiliations:** 1 Department of Pharmacology, Institute of Basic Medical Sciences, Khyber Medical University, Peshawar, Khyber Pakhtunkhwa, Pakistan; 2 Department of Pharmacy, University of Malakand, Chakdara Dir, Khyber Pakhtunkhwa, Pakistan; 3 Department of Pharmacy, Abasyn University, Peshawar, Pakistan

**Keywords:** *Rubus fruticosus*, Anthelmintic, *Ascaridia galli*, *Raillietina spiralis*, Jejunum, Antispasmodic

## Abstract

**Background:**

*Rubus fruticosus* is used in tribal medicine as anthelmintic and an antispasmodic. In the current work, we investigated the anthelmintic and antispasmodic activities of crude methanol extract of fruits of *R. fruticosus* on scientific grounds. Acute toxicity and brine shrimp cytotoxicity activity of the extract were also performed.

**Methods:**

Acute toxicity study of crude methanol extract of *R. fruticosus* was performed on mice. *In vitro* Brine shrimp cytotoxicity assay was performed on shrimps of *Artemia salina. In vitro* Anthelmintic activity was tested against *Raillietina spiralis* and *Ascaridia galli.* Relaxant activities were tested on spontaneous rabbits’ jejunal preparations. Calcium chloride curves were constructed to elucidate possible mode of action of the extract.

**Results:**

LD _50_ of the extract for acute toxicity studies was 887.75 ± 9.22 mg/ml. While CC _50_ of the extract for Brine shrimps cytotoxicity assay was 13.28 ± 2.47 μg/ml. Test samples of crude methanolic extract of *R. fruticosus* (Rf.Cr) at concentration 20 mg/ml showed excellent anthelmintic activity against *Raillietina spiralis.* Anthelmintic activity was 1.37 times of albendazole against the *Raillietina spiralis* at concentration 40 mg/ml. At higher concentration (40 mg/ml), Rf.Cr has 89. 83% parasiticidal activity. The mean EC_50_ relaxation activity for spontaneous and KCl-induced contractions was 7.96 ± 0.1 and 6.45 ± 0.29 mg/ml, respectively. EC _50_ (Log[Ca^++^]M) for control calcium chloride curves was −1.75 ± 0.01 *vs*. EC _50_ −1.78 ± 0.06 in the presence of 3.0 mg/ml of Rf.Cr. Similarly, EC _50_(Log[Ca^++^]M) in the absence and presence of verapamil (0.1 μM) were −2.46 ± 0.01 and −1.72 ± 0.02, respectively.

**Conclusions:**

The anthelmintic and relaxant activities explained traditional uses of *R. fruticosus* on scientific grounds. Relaxant activity follows the inhibition of voltage gated channels. Although the plant extract has cytotoxic effects, yet it is evident from acute toxicity study that it is safe in concentration 100 mg/kg. Further work is required to isolate pharmacologically active compounds.

## Background

There are 6000 species of plants occurring in Pakistan that indicate its potential wealth. 700 species of these are used for their medicinal values, while several of them are exported [1]. Several hundred species are used in herbal remedies, in indigenous system of medicine, because of their medicinal importance that may be attributed to their phytochemicals [2]*.*

*R. fruticosus* is locally known as “Karwara” [3]. It grows mostly in Northern areas of Pakistan like Malakand, Kotli, Chitral and Dir districts of Khyber Pakhtunkhwa. The plant is scrambling perennial shrub, which belongs to family Rosaceae. The family is largest dicot family of vascular plants which comprises 85 genera and 3000 species, all of which are mostly located in tropics to alpine pastures of Pakistan as evergreen or deciduous trees, shrubs and herbs [4].

*R. fruticosus* is a perennial shrub, with spiny branches*,* leaves tri or penta foliate; leaflets leathery, elliptic or obviate, toothed, green above, grey-woolly beneath, the terminal largest; leaf stalk with long bristles; flowers white, in dense short branched cluster and fruits are black.

It contains ascorbic acid, organic acids, tannins and volatile oils [5]. On the basis of these chemical constituents, the plant is very useful antidiarrheal and soothes inflamed mucosa. Decoction of leaves is used as tonic and gargle. Poultice of the leaves is applied to abscesses and skin ulcers [6,7].

Its ethno-medical and biological activities include anti-inflammatory, antimicrobial and antioxidant. The plant is traditionally used in the treatment of wound healing, dysentery, diarrhoea, haemorrhoids, cystitis and diabetes mellitus [8]. Since the plant has potential medicinal properties as antispasmodic and anthelmintic, therefore, we conducted current work to investigate: 1) the antispasmodic activity of *R. fruticosus* in the context of abdominal pain, and 2) the anthelmintic activity of *R. fruticosus*.

## Methods

### Collection, authentication and extraction of plant materials

Mature fruits of *R. fruticosus* were collected from the nearby hilly areas of University of Malakand in the month of July, 2008. The plant was identified by plant taxonomist Professor Dr. Jehandar Shah. A voucher specimen (Rf-01-2008) was submitted to the herbarium of University of Malakand. The fruits (0.8 kg) were dried in shade, pulverized and macerated in hydro methanolic mixture (70%) for 5 days. The materials were filtered off. The filtrate was combined and concentrated using rotary evaporator at controlled temperature 40–45°C. A dark brownish extract 30 g was obtained.

### Preliminary phytochemical screenings

The plant materials were tested for the presence of alkaloids, flavonoids, tannins, saponins, glycosides, terpenoids, sterols and carbohydrates [9-11].

### Solvents, chemicals, drugs and animals

Commercial grade solvents were double distilled before its use in the experiments. Analytical grade (Merck grade) chemicals were used in the experiments. All solutions were prepared on the same day of experiments. Acetylcholine was purchased from Poole chemicals, UK. Rests of the chemicals were purchased from Merck. Rabbits (either sex) were purchased from the local market. Their average weight was in range of 1.5-2.5 kg. They were housed in the animal house of University of Malakand and Khyber Medical University, Peshawar. Roundworms and tapeworms were obtained from the intestines of freshly slaughtered fowls. All fecal matters were removed from intestines with help of normal saline solution. The worms were collected after dissection of intestines and maintained in normal saline solution, having an average size of roundworms and tapeworms as 5–7 cm and 6–7 cm, respectively. Zoologist at department of Zoology, University of Malakand identified the parasites. Experimental protocols were in compliance with “Animals Byelaws 2008 of the University of Malakand”. Ethical Committee of Department of Pharmacy, University of Malakand approved the experimental protocols.

### Interpretation of data and statistical analysis

Chart 7, supplied with the Power Lab, was used to interpret the data. Statistical analysis was performed at 95% confidence interval. *P* value equal to or less than 0.05 was considered as significant. Graph Pad prism was used to calculate mean, SEM and draw the curves for EC_50_ shift.

### Acute toxicity studies

Acute toxicity studies were performed in two phases in mice. Mice of either sex were fasted overnight. The crude methanol extract was administered intraperitoneally (i.p). In phase I, acute toxicity studies of test samples were performed in concentrations 10, 100, 1000 mg/kg (I.p) to determine lethal range. Six mice were in each test group. In the second phase, we conducted the study in concentrations 750, 1000 and 1250 mg/kg in test animals. Numbers of deaths were recorded within 24 hours. Per cent lethality was noted [12].

### Brine shrimp cytotoxicity

Brine shrimps’ eggs were hatched in laboratory at room temperature. 10 shrimps, 5 ml sea water and extract in concentrations 10, 100 and 1000 ppm were added to a vial. An incubation period of 24 hours was given at room temperature. Visual counting of dead brine shrimps (laying at bottom) was performed. Per cent cytotoxicity was determined [13].

### Anthelmintic activity

Anthelmintic activity was performed against test parasites round worms (*Raillietina spiralis*) and tape worms (*Ascaridia galli*). The parasites were obtained from the intestine of infested fowls obtained from the nearby slaughter house of Chakdara. They were maintained in distilled water in petri dishes at room temperature. Tests solutions of *R. fruticosus* were prepared in concentrations of 10, 20 and 40 mg/ml. Approximately 25 ml of respective solutions along with parasites of equal size were kept in the petri dishes. Time for paralysis (anthelmintic activity) was recorded when no movement was observed except when shaken vigorously. Time for death (parasiticidal activity) was recorded when the worms did not show any movement by vigorous shaking nor when dipped in warm water (50°C). Distilled water was used as negative control. Albendazole and piperazine citrate were used as positive controls [14-17].

### Effects on spontaneous rabbits’ jejunum preparations and KCl induced contractions

Rabbits of either sex were subjected to cervical dislocation. Their abdomens were opened and pieces of jejunums were removed. Jejunal preparations were maintained in Tyrode’s solution, which was constantly aerated with carbogen gas (95% oxygen: 5% carbon dioxide mixture). The tissues were freed from mesentery. The concentrations of constituents of Tyrode’s solution were (mM): KCl 2.68, NaCl 136.9, MgCl_2_ 1.05, NaHCO_3_ 11.90, NaH_2_ PO_4_ 0.42, CaCl_2_ 1.8 and glucose 5.55. Preparations of about 1.3 - 1.5 cm lengths were mounted in 10 ml tissue bath containing Tyrode’s solution. Temperature was maintained as 37 ± 1°C. After stabilization, the test samples were tried in concentrations 0.01, 0.03, 0.1, 0.3, 1.0, 3.0,5 and 10 mg/ml. Force Transducer (Model No: MLT 0210/A Pan Lab S.I.) connected with Power lab (Model No: 4/25 T) was used to record intestinal responses.

Similarly, sustained contractions were produced by 80 mM solution of KCl in the rabbits’ jejunal preparations. Test samples were tested in similar concentrations to determine its possible mode of action through calcium channels [18-21].

### Effects on calcium chloride curves

Since the test extract produced concentration dependent relaxation on KCL-induced contractions, hence, we constructed calcium chloride curves to search for its possible mode of action. Tissues were decalcified by K-Normal and K-rich solution thereafter. Constituents and concentration (mM) of K-Rich Tyrode’s solution was KCl 50, NaCl 91.04, MgCl_2_ 1.05, NaHCO_3_ 11.90, NaH_2_ PO_4_ 0.42, glucose 5.55 and EDTA 0.1. Earlier the tissues were stabilized in normal Tyrode’s solution. Control Calcium Chloride Curves (CCs) were constructed twice. Control maximum was used as standard curve for comparison. The CCs were constructed in the presence of test samples where an incubation period of one hour was given. Effects on EC_50_ were compared with respective control. Similarly, curves for verapamil, a standard calcium channel blocker, were constructed. Effects on EC_50_ were noted and compared for possible right shift [22-24].

## Results and discussion

**Figure 1:**
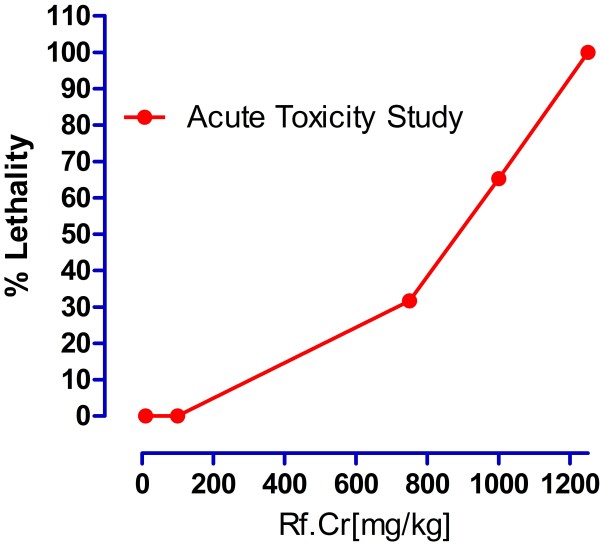
**Acute toxicity of crude methanol of *R. fruticosus.***

**Figure 2:**
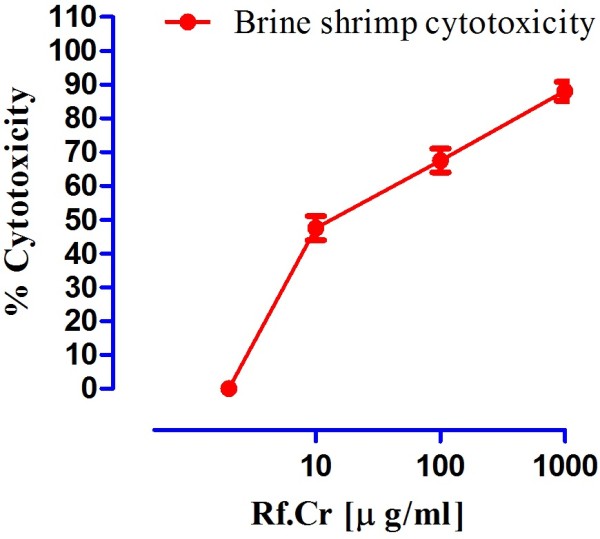
**Brine shrimp cytotoxicity of crude methanol of *R. fruticosus.***

**Figure 3:**
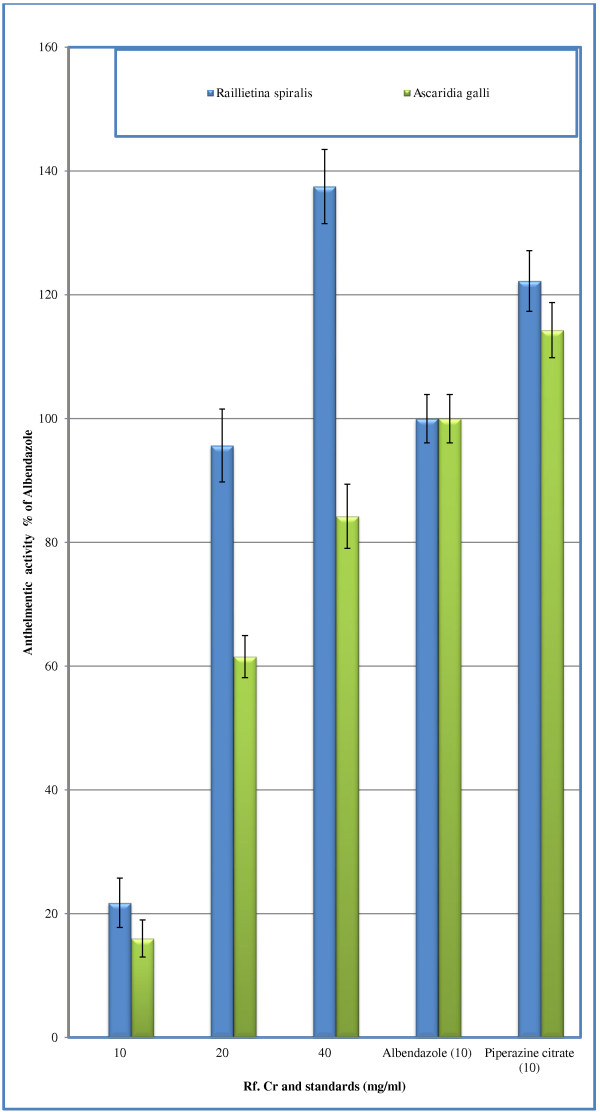
**Anthelmintic activity of *R. fruticosus *and standard drugs.** (*n =* 3, per cent expression is based on paralysis data).

**Figure 4:**
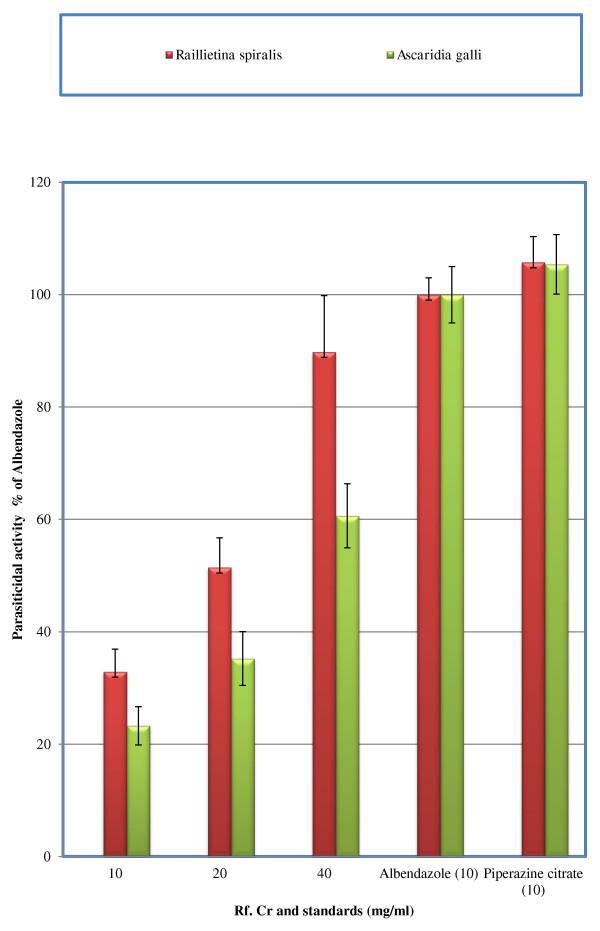
**Parasiticidal activity of *R. fruticosus *and standard drugs as per cent of albendazole 10 mg/ml, *n = *3, per cent expression is based on death of test parasites.**

The test samples tested strongly positive for the presence of saponins. It also tested positive for the presence of flavonoids, tannins, sesquiterpenes, proteins and carbohydrates. It tested negative for terpenoids. Results of acute toxicity studies are expressed in Figure 1. The LD_50_ is 887.75 ± 9.2 (*n* = 4) mg/kg. It is concluded that the extract is safe up to 100 mg/kg. Results of Brine shrimp cytotoxicity are expressed in Figure 2. Where the CC_50_ is 13.275 ± 2.47 μg/ml. It reveals that the plant extract carries cytotoxic activity and can be good source of cytotoxic compounds because of the previous literature reports for positive correlation between the brine shrimp cytotoxicity assay and human KB cell line (human nasopharyngeal carcinoma) [25]. Thus the current work warrants for isolation of these anticancer constituents present in the plant [26]. Results of anthelmintic activity are shown in Table 1. Time taken for paralysis and death are expressed in minutes. Test samples of crude methanolic extract of *R. fruticosus* at concentrations 20 mg/ml showed excellent anthelmintic activity against *Raillietina spiralis.* Anthelmintic activity is 1.37 times of albendazole against the said parasite at concentration 40 mg/ml (Figure 3). This strongly suggests its traditional use as anthelmintic. Against *Ascaridia galli,* anthelmintic activity is 61.5% and 84.2% at concentrations 20 and 40 mg/ml respectively. However, parasiticidal activity, as per cent of albendazole (10 mg/ml), is expressed in Figure 4. At higher concentration (40 mg/ml), Rf.Cr has 89. 83% parasiticidal activity. It is important to note that this parasiticidal activity is percent of albendazole, a standard parasiticidal drug. Hence, the results confirm its traditional use as anthelmintic as per previous reported work. The anthelmintic activity may be attributed to the phytochemicals particularly the flavonoids, saponins, tannins and sesquiterpenes as similar types of compounds have been proven to be anthelmintic [14-17].

**Table 1:** **Anthelmintic activity of *R. fruticosus *against *Raillietina spiralis *and *Ascaridia galli***

**Test samples and standards**	**Conc. (mg/ml)**	***Raillietina spiralis***		***Ascaridia galli***	
		**P**	**D**	**P**	**D**
*R. fruticosus*	10	101	161	100	159
	20	23	103	26	105
	40	16	59	19	59
Albendazole	10	22	55	16	39
Piperazine citrate	10	18	52	14	37
Negative control	-	-	-	-	-

**Figure 5:**
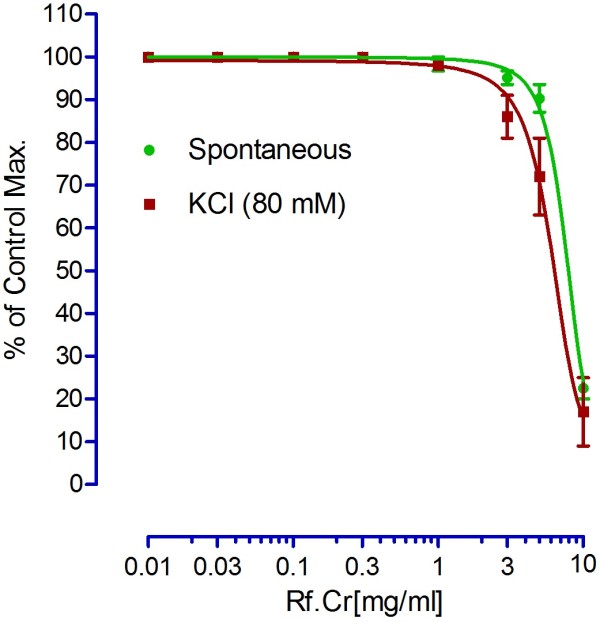
**The effects of crude methanol extract on spontaneous and KCl-induced contractions (Values are mean ± SEM, *n *= 4, *P *< 0.05).**

**Figure 6:**
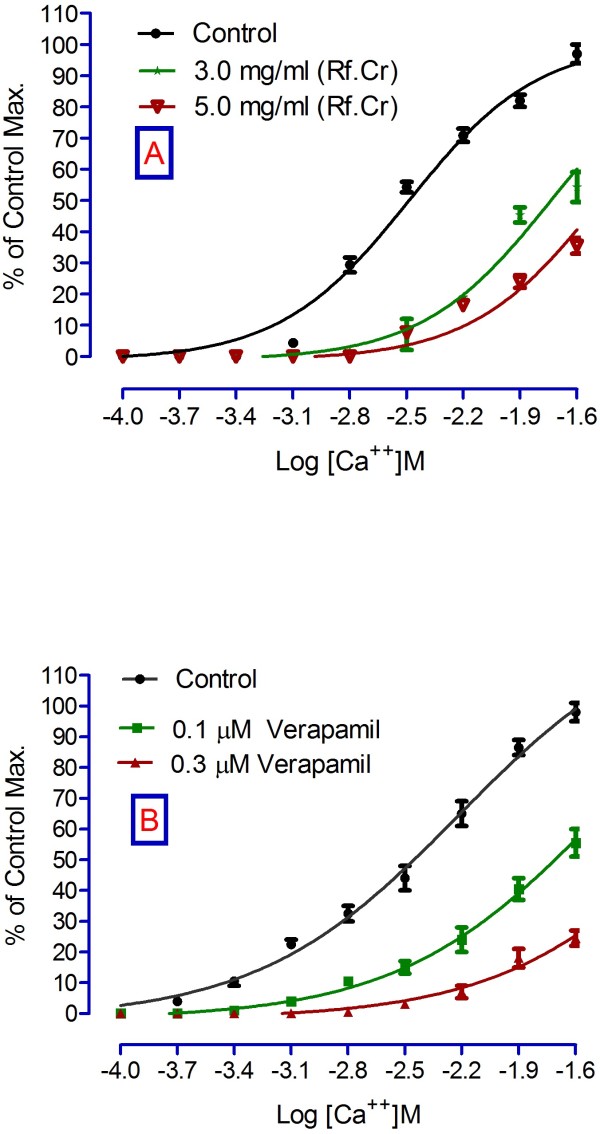
**Constructing calcium chloride curves: A) in the absence (control) and presence of test samples. B**) in the absence and presence of verapamil (Values are mean ± SEM, *n* = 4, *P* < 0.05).

Relaxant activity on spontaneous rabbits’ jejunal preparations and 80 mM KCl-induced contractions are expressed in Figure 5. It relaxed the spontaneous contractions and showed a concentration dependent relaxing effect on jejunal preparations. Many mechanisms are sometimes involved in relaxing effect. Like relaxing effects may be either through calcium antagonistic action or through blocking of muscarinic receptors or both. In addition, there may be involvement of histaminergic receptors. Nevertheless, relaxing effects on KCl-induced contractions provides a rapid screening for possible relaxing mechanisms that may involve voltage gated calcium channels [21-23]. Voltage gated channels play a vital role in the regulation of peristaltic movements of the intestine as it helps in periodic depolarization and repolarization of the gastrointestinal tract [23,24]. Since KCl induces contractions via calcium influx from extracellular medium to intracellular medium, hence, relaxing effects on KCl-induced contractions may be regarded to follow calcium channel blocking mechanism [19]. EC_50_ for spontaneous and KCl- induced contractions are 7.96 ± 0.1 and 6.45 ± 0.29 mg/ml. To confirm its possible mechanism through voltage gated channels, we constructed calcium chloride curves (CCs) in K-rich medium. CCs in the absence and presence of the test sample are expressed in Figure 6. According to Figure 6A, EC _50_ (Log[Ca^++^]M) for control calcium chloride curves is −1.75 ± 0.01 *vs*. EC _50_ -1.78 ± 0.06 in presence of 3.0 mg/ml. Similarly, EC _50_ (Log[Ca^++^]M) in the absence and presence of verapamil (0.1 μM) are −2.46 ± 0.01 and −1.72 ± 0.02, respectively (Figure 6B). Thus the right shift produced by Rf.Cr resembles the right shift of verapamil, a calcium channel blocker. Hence, we conclude that the mode of jejunal relaxation observed might be mediated by voltage gated calcium channels. The activities are attributed to the phytochemicals that are present in the extract.

## Conclusions

The anthelmintic and relaxant activities explain traditional uses of *R. fruticosus* on scientific grounds. Relaxant activity follows the inhibition of voltage gated channels. Although the plant extract has cytotoxic effects, yet it is evident from acute toxicity study that it is safe at concentration 100 mg/kg. Further work is required to isolate the pharmacologically active compound(s).

## Abbreviations

Rf.Cr: Crude methanolic extract of *Rubus fruticosus*.

## Competing interests

The authors declared that they have no competing interests.

## Authors’ contributions

NA participated in collection of CCs data, interpretation and writing the manuscript. UA M. Phil research scholar and collected the data. SWA helped in acute toxicity studies. IS helped in preparation of the extract, and literature survey. MJ helped in literature survey and phytochemistry of test samples. GA helped in construction of CCs. WA helped in cytotoxicity studies. MG helped in animal breeding. All authors read and approved the manuscript.

## Pre-publication history

The pre-publication history for this paper can be accessed here:

http://www.biomedcentral.com/1472-6882/13/138/prepub
